# Network‐based computational approach to identify genetic links between cardiomyopathy and its risk factors

**DOI:** 10.1049/iet-syb.2019.0074

**Published:** 2020-04-01

**Authors:** Md. Nasim Haidar, M. Babul Islam, Utpala Nanda Chowdhury, Md. Rezanur Rahman, Fazlul Huq, Julian M.W. Quinn, Mohammad Ali Moni

**Affiliations:** ^1^ Department of Electrical and Electronic Engineering University of Rajshahi Rajshahi 6205 Bangladesh; ^2^ Department of Computer Science and Engineering University of Rajshahi Rajshahi 6205 Bangladesh; ^3^ Department of Biochemistry and Biotechnology School of Biomedical Science, Khwaja Yunus Ali University Sirajgonj 6751 Bangladesh; ^4^ School of Medical Sciences Faculty of Medicine and Health The University of Sydney Sydney NSW 2006 Australia; ^5^ Bone Biology Division Garvan Institute of Medical Research Darlinghurst NSW 2010 Australia

**Keywords:** cellular biophysics, diseases, molecular biophysics, proteins, RNA, neurophysiology, bioinformatics, medical disorders, biochemistry, data analysis, biology computing, genomics, genetics, network‐based computational approach, cardiomyopathy, lifestyle factors, inflammatory CMP development, systems biology approach, microarray gene expression datasets, risk factors including smoking, ageing factors, clinical depression status, high dietary red meat intake, high‐calorie diet, high‐fat diet, differentially expressed genes, risk factor datasets, protein–protein interaction network analysis identified protein subnetworks, CDT1, HIST1H4C, HIST1H4D, transcription factors, FOXC1, FOXL1, YY1, CREB1, authors, important risk factors, managing CMP

## Abstract

Cardiomyopathy (CMP) is a group of myocardial diseases that progressively impair cardiac function. The mechanisms underlying CMP development are poorly understood, but lifestyle factors are clearly implicated as risk factors. This study aimed to identify molecular biomarkers involved in inflammatory CMP development and progression using a systems biology approach. The authors analysed microarray gene expression datasets from CMP and tissues affected by risk factors including smoking, ageing factors, high body fat, clinical depression status, insulin resistance, high dietary red meat intake, chronic alcohol consumption, obesity, high‐calorie diet and high‐fat diet. The authors identified differentially expressed genes (DEGs) from each dataset and compared those from CMP and risk factor datasets to identify common DEGs. Gene set enrichment analyses identified metabolic and signalling pathways, including MAPK, RAS signalling and cardiomyopathy pathways. Protein–protein interaction (PPI) network analysis identified protein subnetworks and ten hub proteins (CDK2, ATM, CDT1, NCOR2, HIST1H4A, HIST1H4B, HIST1H4C, HIST1H4D, HIST1H4E and HIST1H4L). Five transcription factors (FOXC1, GATA2, FOXL1, YY1, CREB1) and five miRNAs were also identified in CMP. Thus the authors’ approach reveals candidate biomarkers that may enhance understanding of mechanisms underlying CMP and their link to risk factors. Such biomarkers may also be useful to develop new therapeutics for CMP.

## 1 Introduction

Cardiomyopathy (CMP) is a group of diseases affecting the structure and functioning of the heart, and includes conditions where the heart is affected by ventricular hypertrophy, dilation or fibrotic dysplasia that cause mechanical and electrical dysfunction. CMP may be either cardiac‐specific or a part of generalised systemic disorders, but many of these conditions result in cardiovascular damage or progressive heart failure [1]. CMP is the third most prevalent cause of heart failure in the USA [1]. In 2015, about 2.6 million people worldwide were affected by cardiomyopathy and myocarditis [2]. Currently, the most commonly occurring form of CMP is dilated CMP which affects five in 100,000 adults and 0.57 in 100,000 children [3].

The etiology of the cardiomyopathy involves genetic, infectious, metabolic and environmental factors [1]. Lifestyle risk factors include severe obesity, alcohol consumption (AC), long‐term high blood pressure, coronary heart disease, and sarcoidosis, but the molecular mechanisms behind the development of CMP and how these risk factors contribute to the progression of the CMP is not well understood. However, we can use our knowledge of CMP risk factors to identify key factors in CMP development by determining the altered gene expression patterns the risk induces that are also seen CMP‐affected heart tissues. Using an integrative gene‐network‐based approach we can then identify candidate causative pathways that can be further examined [4, 5].

**Fig. 1:**
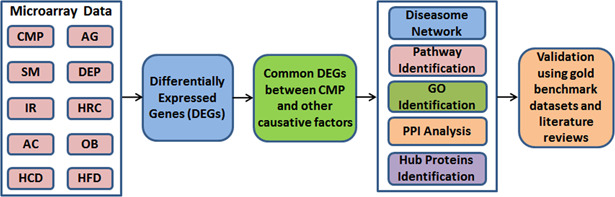
Overview of the quantitative network‐based approach employed in this study. The microarray gene expression datasets of CMP (GSE4172), ageing (GSE1144), smoking (GSE4806), depression (GSE12654), high RM consumption (GSE25220), IR (GSE20950), chronically high AC (GSE44456), morbid obesity (GSE48964), HCD (GSE56960) and HFD datasets (GSE68231) have been obtained from NCBI‐GEO

Integrative network‐based gene or multi‐omics analyses are an increasingly common approach used to identify disease‐associated biomarkers and therapeutic targets [6]. Such an approach is now commonly used for elucidating molecular mechanisms in different disease such as Alzheimer's disease [7, 8, 9, 10, 11, 12], Parkinson's disease [13, 14, 15], multiple sclerosis [16], respiratory system diseases [17], colorectal cancer [18] and Thyroid cancers [19, 20, 21]. Therefore, in this study, a system biology‐based approach was used to identify molecular biomarker transcripts (i.e. mRNAs), and proteins (hub proteins) and pathways in CMP using CMP‐associated risk factors to clarify the genes that may be causative factors for the progression of CMP (Fig. 1). For this purpose, we first identified DEGs, genes whose expression is altered in CMP affected tissues and in risk‐factor exposed tissues; these DEGs that were common between CMP and particular CMP‐associated risk factors were then identified. These common DEGs, were then studied for their involvement in human biomolecular networks such as protein–protein interaction (PPI) networks to identify central signalling molecules (hub proteins) and molecular pathways. This resulted in the identification of candidate genes that could mediate influences of the CMP risk factors, and these were then cross‐validated using gold benchmarking datasets OMIM and dbGaP gene‐disease association databases to identify those candidates with known pathological involvement.

## 2 Materials and methods

### 2.1 High‐throughput microarray gene expression datasets

We analysed gene expression microarray datasets to identify the molecular association of different factors with CMP at the molecular level. All the datasets used in this study were collected from the National Center for Biotechnology Information (NCBI) Gene Expression Omnibus [22], and employed Affymetrix Human DNA arrays unless otherwise stated. The utilised gene expression datasets with accession numbers GSE4172, GSE1144, GSE4806, GSE12654, GSE20950, GSE25220, GSE44456, GSE48964, GSE56960 and GSE68231 were analysed in this study. The CMP dataset (GSE4172) was obtained by gene expression profiling of human inflammatory CMP [23]. The ageing (AG) dataset (GSE1144) was obtained by analysing gene expression in skeletal muscle tissue characterised by loss of metabolic and contractile competence [24]. The smoking (SM) dataset (GSE4806) was obtained from gene expression profiles of T‐lymphocytes from smokers and non‐smokers [25]. The depression (DEP) dataset (GSE12654) was attained by gene expression from the human prefrontal cortex (BA10) [26]. The dataset (GSE20950) for insulin resistance (IR) was obtained by gene expression data from human adipose tissue using an IR patient cohort [27]. The red meat (RM) dietary intervention dataset (GSE25220) was generated using an Agilent‐014850 whole human genome microarray data from human colon biopsies before and after participating in a high RM dietary intervention [28]. The AC dataset (GSE44456) was obtained by examining gene expression in post‐mortem hippocampus tissues from 20 alcoholics and 19 controls [29]. The obesity (OB) dataset (GSE48964) was obtained by expression data from adipose stem cells (ASCs) from morbidly obese and non‐obese individuals [30]. The high‐calorie diet (HCD) dataset (GSE56960) was obtained by expression profiling of array of blood cell transcriptome of two different population groups after the ingestion of different caloric doses [31]. The high‐fat diet (HFD) dataset (GSE68231) is Affymetrix Human Genome data obtained from human skeleton muscle of five subjects in each group selected before and after three days of an HFD [32].

### 2.2 Identification of differentially expressed genes

We performed a differential gene expression analysis of the CMP with nine risk factors from transcriptomics datasets. Firstly, we transformed each gene expression data for each disease using the *Z*‐score (or zero mean) normalisation method for both disease and control states. This might resolve the problems regarding mRNA data comparisons using different platforms and experimental set‐ups [33]. Each sample of the gene expression matrix was normalised using mean and standard deviation. The expression value of the gene *i* in sample *j* represented by $g_{ij}$ was transformed into $Z_{ij}$ by computing

(1) $$Z_{ij}=\frac{g_{ij}-\text{mean}(g_{i})}{\text{SD}(g_{i})}$$

where SD is the standard deviation. Comparing values of gene expression for various samples and diseases are made possible by this transformation.

The gene expression datasets were normalised by $\log_{2}$ transformation and unpaired student *t*‐test was used. Finally, genes were filtered by setting threshold values with adjusted *p*‐value <0.05 and absolute log fold change (log FC) >1.0 to designate statistically significant DEGs.

### 2.3 Gene set enrichment analysis to identify gene ontology and pathways

To clarify the biological significance of the identified DEGs, gene‐set enrichment analysis and pathways analysis were performed to identify the significant gene ontology terms and KEGG pathways enriched by DEGs via EnrichR [34, 35]. For statistical significance, the adjusted *p*‐value < 0.05 was considered for the significance assessment of enrichment results.

### 2.4 Identification of transcriptional and/or post‐transcription regulators of the DEGs

To identify regulatory transcription factors (TFs) that regulate the DEGs at the transcriptional level, TF‐target gene interactions were obtained from the JASPAR database to identify TFs based on topological parameters [36]. The regulatory miRNAs which regulate DEGs at the post‐transcriptional level were identified from miRNAs‐target gene interactions were obtained from TarBase and miRTarBase based on topological parameters [37, 38, 39].

### 2.5 PPI analysis to identify hub proteins

We reconstructed a PPI network around the proteins encoded by the DEGs using protein interactome database STRING [40]. The PPI network was analysed by Cytoscape (v3.5.1) [41, 42]. An undirected graph representation was used for the PPI network, where the nodes indicate proteins and the edges symbolised the interactions between the proteins. We performed a topological analysis using Cyto‐Hubba plugin [43, 44] in Cytoscape to identify highly connected proteins (i.e. hub proteins) in the network and the degree metrics were employed [45, 46].

### 2.6 Protein–drug interactions analysis

The protein–drug interactions were analysed using the DrugBank database (Version 5.0) to identify potential small molecules that can affect pathways we identified as important in CMP and which may point to therapeutic approaches for CMP [47].

## 3 Results

### 3.1 Identification of differentially expressed genes from microarray gene expression datasets

The gene expression datasets of CMP were analysed and a total of 1764 DEGs were identified in CMP patients compared to control samples where 919 genes were up‐regulated and 845 genes were down‐regulated.

For the investigation of the relationship of the CMP with nine risk factors, we performed several steps of statistical analysis for mRNA microarray data of each risk factor. Thus, we selected the most significant over and under‐regulated genes for each risk factor. Our analysis identified a large number of dysregulated genes. The 1122, 400, 72, 356, 482, 25, 157, 255, 739 DEGs were identified in AG, SM, DEP, IR, RM, AC, OB, HCD, HFD datasets. Then, a cross‐comparative analysis revealed the common over and under‐expressed genes between CMP and above‐mentioned risk factors. The CMP shared 48, 32, 3, 28, 29, 2, 6, 7 and 81 significant DEGs with AG, SM, DEP, IR, RM, AC, OB, HCD and HFD, respectively.

**Fig. 2:**
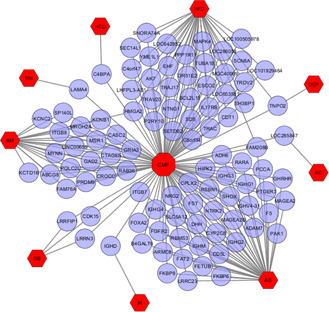
Network for up‐regulated genes of CMP with ageing (AG), smoking (SM), AC, HCD, HFD, IR, obesity (OB), depression (DEP) and high RM intake. The red coloured octagon‐shaped node at the centre represents the target CMP and the hexagon‐shaped nine nodes represent the risk factors and the other nodes represent the genes that are in common between CMP with the examined risk factors

**Fig. 3:**
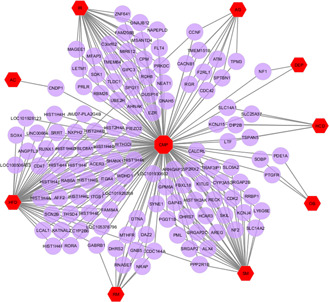
Network for down‐regulated genes of CMP with ageing (AG), smoking (SM), AC, HCD, HFD, IR, obesity (OB), depression (DEP) and high RM intake. The red coloured octagon‐shaped node at the centre represents the target CMP and the hexagon‐shaped nine nodes represent the risk factors and the other nodes represent the genes that are common to CMP and the risk factors

The diseasome association networks centred on the CMP were built to identify statistically significant associations among these risk factors (Figs. 2 and 3 and Table 1). Notably, FAM208B was commonly dysregulated for CMP, AG, HFD and IR. CMP shared 18 DEGs (ESCO2, HIST1H4A, HIST1H4B, HIST1H4C, HIST1H4D, HIST1H4E, HIST1H4F, HIST1H4H, HIST1H4I, HIST1H4J, HIST1H4K, HIST1H4L, HIST2H4A, HIST2H4B, HIST4H4, ITGB1, PIEZO2, SCN8A) with HFD. Three DEGs FGFR2, GHRHR and SPTBN1 were common among CMP, AG. CMP shared GABRB1 and HMGA2 dysregulated genes with HFD and RM. Moreover, CMP and HFD shared CCNF, TNPO2 and PPP2R1B dysregulated genes with AG, DEP and SM, respectively.

**Table 1:** Significant Gene Ontology terms related to CMP and risk factors such as smoking, IR, AC, high caloric diet, high RM intake, depression, HFD, obesity and ageing

Category	GO ID	Term/pathway	Genes	Risk factors	*P*‐value
gene ontology biological process	GO:2000146	negative regulation of cell motility	NF1, SRGAP2C, SRGAP2, NF2, RECK, SRGAP2B	DEP, SM	4.62×10^−7^
	GO:0070828	heterochromatin organisation	MTHFR, HMGA2	HFD, RM	9.07×10^−5^
	GO:2000257	regulation of protein activation cascade	IGHG3, IGHG4, IGHG1, IGHG2, C4BPA	AG, HCD	1.35×10^−4^
	GO:0030334	regulation of cell migration	NF1, SRGAP2C, SRGAP2, NF2, RECK, SRGAP2B	DEP, SM	1.37×10^−4^
	GO:0030449	regulation of complement activation	IGHG3, IGHG4, IGHG1, IGHG2, C4BPA	AG, HCD	1.40×10^−4^
	GO:0002920	regulation of humoral immune response	IGHG3, IGHG4, IGHG1, IGHG2,C4BPA	AG, HCD	1.61×10^−4^
	GO:0002697	regulation of immune effector process	IGHG3, IGHG4, IGHG1, IGHG2, C4BPA	AG, HCD	1.66×10^−4^
	GO:0002673	regulation of acute inflammatory response	IGHG3, IGHG4, IGHG1, IGHG2, C4BPA	AG, HCD	2.08×10^−4^
	GO:0070613	regulation of protein processing	IGHG3, IGHG4, IGHG1, IGHG2, C4BPA	AG, HCD	2.58×10^−4^
	GO:1902531	regulation of intracellular signal transduction	CDC42, PAK1, F2RL1, ATM, HIPK2, CDK2, NF2, SRGAP2, PML, ARHGAP35	AG, SM	5.20×10^−4^
gene ontology	GO:0005887	integral components of plasma membrane	SLC14A1, KCNJ15, TSPAN5, PTGFR	HCD, OB	1.10×10^−2^
	GO:0030424	axon	NTRK2, PAK1, NF1,	AG, DEP, RM	1.79×10^−2^
cellular component			KCNB1, KCNC2		
	GO:0071437	invadopodium	PAK1, EZR	AG, IR	1.81×10^−2^
	GO:0030425	dendrite	KCNB1, KCNC2, NF1	DEP, RM	3.21×10^−2^
	GO:0005856	cytoskeleton	CDC42, TPM3, RARA,	AG, OB	3.60×10^−2^
			SPTBN1, LRRFIP1		
gene ontology molecular function	GO:0005096	GTPase activator activity	SRGAP2C, SRGAP2, NF1, ARHGAP35, SRGAP2B	SM, DEP	6.51×10^−4^
	GO:0030695	GTPase regulator activity	SRGAP2C, SRGAP2, NF1, ARHGAP35, SRGAP2B,	SM, DEP	9.41×10^−4^
	GO:0015204	urea transmembrane transporter activity	SLC14A1, SLC14A2	DEP, HCD, SM	1.05×10^−3^
	GO:0042887	amide transmembrane transporter activity	SLC14A1, SLC14A2	DEP, HCD, SM	1.35×10^−3^
	GO:0004955	prostaglandin receptor activity	PTGFR, PTGER3	OB, AG	3.30×10^−3^
	GO:0015467	G‐protein activated inward rectifier potassium channel activity	KCNJ15, KCNJ4	HCD, SM	3.84×10^−3^
	GO:0022838	substrate‐specific channel activity	SLC14A1	DEP, HCD	4.19×10^−3^
	GO:0005249	voltage‐gated potassium channel activity	KCNB1, KCNC2, KCNJ15	RM, HCD	6.88×10^−3^
	GO:0005242	inward rectifier potassium channel activity	KCNJ15, KCNJ4	HCD, SM	7.68×10^−3^
	GO:0003680	AT DNA binding	HMGA2	RM, HFD	1.15×10^−2^

### 3.2 Molecular pathway and functional analysis

To clarify the biological roles of the identified common DEGs between CMP and other risk factors, we performed gene ontology analysis to identify the biological process, cellular component and molecular functions enriched by the DEGs (Table 2). There was a total of 741 significant gene ontology groups including leukocyte adhesion to vascular endothelial cell (GO:0061756), cellular response to reactive nitrogen species (GO:1902170), mesoderm formation (GO:0001707), cAMP‐mediated signalling (GO:0019933), morphogenesis of an epithelium (GO:0002009), negative regulation of response to biotic stimulus (GO:0002832), leukocyte tethering or rolling (GO:0050901), establishment of epithelial cell apical/basal polarity (GO:0045198), regulation of osteoblast proliferation (GO:0033688), mesodermal cell differentiation (GO:0048333) were identified.

**Table 2:** Some significant KEGG pathways those are common among inflammatory CMP and other risk factors such as SM (smoking), IR, AC, HCD (high caloric diet), RM (high red meat intake), depression, HFD (high fat diet), obesity and ageing

KEGG ID	Pathway	Genes in pathway	Risk factors
hsa00410	beta‐Alanine metabolism	CNDP1, GAD2	AC, RM
hsa04010	MAPK signalling pathway	CACNB1, CDC42, NTRK2, PAK1, FGFR2, NF1	AG, DEP
hsa04014	RAS signalling pathway	CDC42, PAK1, FGFR2, NF1	AG, DEP
hsa04510	focal adhesion	CDC42, PAK1, ITGB7, LAMA4, ARHGAP35	AG, SM
hsa05032	morphine addiction	PDE1A, GABRB1, GNB5	OB, RM
hsa05200	pathways in cancer	CDC42, TPM3, PTGER3, RARA, FGFR2, KITLG, LAMA4, CDK2, PML	AG, SM
hsa05202	transcriptional misregulation in cancer	RARA, ATM, ITGB7, HMGA2, GRIA3	AG, RM
hsa05410	hypertrophic cardiomyopathy (HCM)	CACNB1, TPM3, ITGB7, ITGB1, ITGA4, SLC8A1	AG, HFD
hsa05412	arrhythmogenic right ventricular cardiomyopathy (ARVC)	CACNB1, ITGB7, ITGB1, ITGA4, SLC8A1	AG, HFD
hsa05414	dilated cardiomyopathy	CACNB1, TPM3, ITGB7, ITGB1, ITGA4, SLC8A1	AG, HFD

The significantly altered molecular pathways were identified in CMP and other risk factors. A total of 61 pathways were found to be over‐represented among several groups out of which some significant pathways are shown in Table 2. The amino acid metabolism pathway such as alanine metabolism pathways, different signalling pathways such as MAPK and RAS signalling pathways, ECM pathways, and alcoholism came into prominence as signalling pathways.

### 3.3 Identification of regulatory biomolecules

**Fig. 4:**
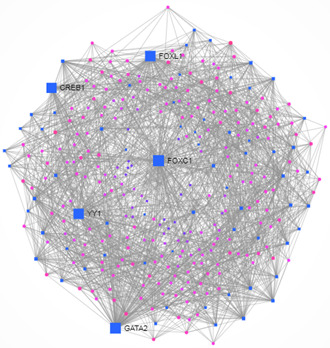
Differentially expressed genes and TF interactions were analysed to identify the TFs that regulate the differentially expressed genes in CMP

**Fig. 5:**
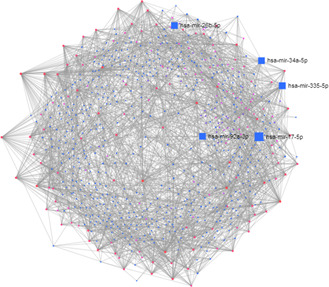
Differentially expressed genes and microRNAs interactions were analysed to identify the microRNAs that regulate the differentially expressed genes in CMP

We studied the regulators of the common DEGS utilising DEGs–TFs and DEG–miRNAs interaction analyses, presented in Table 3. We identified DEG–TFs interactions (Fig. 4) and DEGs–miRNAs interactions (Fig. 5) and detected central regulatory biomolecules (TFs and miRNAs) using topological parameters. As shown in Table 3, five TFs (FOXC1, GATA2, FOXL1, YY1, and CREB1) and five miRNAs (mir‐335‐5p, mir‐26b‐5p, mir‐34a‐5p, mir‐92a‐3p, and mir‐17‐5p) were detected from the DEGs–TFs and DEGs–miRNAs interaction networks, respectively. These biomolecules regulate genes at transcriptional and post‐transcriptional levels.

**Table 3:** Summary of transcriptional and/or post‐transcriptional regulators (TFs and microRNAs) of the deferentially expressed genes

	Symbol	Description	Feature
TFs	FOXC1	Forkhead Box C1	play critical role in early cardiomyogenesis [48]
	GATA2	GATA Binding Protein 2	afflicted with early onset familial coronary artery disease [49]
	FOXL1	Forkhead Box L1	associated with good outcome in human pancreatic ductal adenocarcinoma [50]
	YY1	YY1 TF	increased in human heart failure [51]
	CREB1	CAMP Responsive Element Binding Protein 1	cardiac failure is afflicted with CREB [52]
microRNAs	mir‐335‐5p	MicroRNA 335	upregulated in heart failure; involved in hypertrophic cardiomyopathy pathway [53]
	mir‐26b‐5p	MicroRNA 26	associated with suppression of proliferation and enhance the apoptosis in multiple myeloma cells [54]
	mir‐34a‐5p	MicroRNA 34	could prevent autophagic cell deaths in ischaemic hearts and in this way can improve the myocardial injury [55]
	mir‐92a‐3p	MicroRNA 92	increase blood vessel growth and recovery of damaged tissues in myocardial infarction [56]
	mir‐17‐5p	MicroRNA 17	prognostic markers of hepatocellular carcinoma [57]

The FOXC1 is a TF that plays a critical role in early cardiomyogenesis [48]. It is also required for the morphogenesis process of cardiac outflow tract [58]. The TF GATA2 expression is high in the thoracic aorta and GATA2 variants are associated with early‐onset familial coronary artery disease [49]. FOXL1 is a TF whose elevated expression is associated with good outcomes in human pancreatic ductal adenocarcinoma [50] but does not have a known association with cardiac diseases. The activity of YY1 TF is increased in human heart failure [51]. CREB over‐expression is associated with cardiac failure suggesting it plays a significant role in cardiac pathologies [52].

microRNAs (miRNAs) are short single‐stranded RNA molecules (∼22 nucleotides long) that regulate the expression of genes at post‐transcriptional stage. miRNAs are being considered as potential sources of biomarkers for complex disease including neurodegenerative disease and cancers. Therefore, we have identified those miRNAs controlling the DEGs to provide insights into the regulatory biomolecules. Among the miRNAs, mir‐335‐5p was identified as upregulated in experimental heart failure by experimental animals [53]. Sun *et al.* [59] also predicted mir‐335‐5p is implicated in hypertrophic CMP pathway by microarray analysis. Jia *et al.* [54] showed mir‐26b‐5p was associated with suppression of proliferation and enhance the apoptosis in multiple myeloma cells. It has been proposed the mir‐34a‐5p could prevent autophagic cell deaths in ischemic hearts and in this way can improve the myocardial injury [55, 60]. The inhibition of mir‐92a‐3p leads to increase blood vessel growth and recovery of damaged tissues in myocardial infarction mice models, which suggest it may be an important therapeutic target in ischaemic heart disease [56]. The mir‐17‐5p has been suggested as important prognostic biomarkers in cancer, including hepatocellular carcinoma [57].

### 3.4 PPI network analysis

**Fig. 6:**
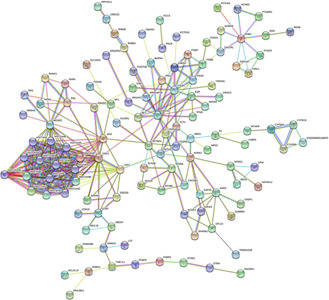
PPI network of differentially expressed genes that were common between CMP and other risk factors

**Fig. 7:**
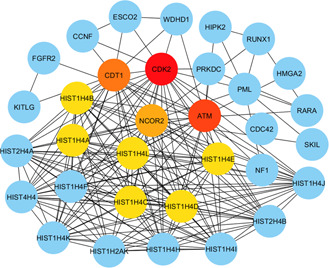
Simplified PPI network of the common differentially expressed genes between CMP and the risk factors. Ten significant hub proteins are marked as red, orange and yellow, respectively

The PPI network was constructed using all the distinct 236 differentially expressed genes that were common between the CMP and the risk factors (Figs. 6 and 7). The topological analysis using degree matrices was used to identify highly connected proteins clusters. Each node in the network represents a protein and an edge indicates the interaction between two proteins. We detected ten hub proteins (CDK2, ATM, CDF1, NCOR2, HIST1H4A, HIST1H4B, HIST1H4C, HIST1H4D, HIST1H4E and HIST1H4L) in PPI analysis. These hub proteins may be potential drug targets.

### 3.5 Identification of candidate drugs

**Fig. 8:**
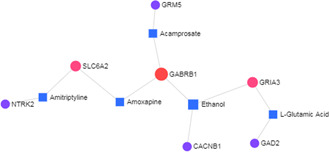
Protein–drug interactions network. The interactions between drugs and the hub node (TUBB) were represented. The area of the node represents the degree of interaction in the network

A protein–drug interaction network was analysed and we found GABRB1, GRIA3, SLC6A2, GAD2, GAD2, CACNB1, NTRK2, and GRM5 proteins had interaction with 5 drugs/compounds (Ethanol, Amoxapine, L‐Glutamic Acid, Amitriptyline, Acamprosate) as shown in Fig. 8.

## 4 Discussion

In this study, the molecular mechanisms that may link CMP and associated risk factors were investigated. We performed an analysis of gene expression data from CMP tissue analysis and from the risk factors in order to identify the common DEGs shared by CMP and the risk factors. This identified CMP affected tissues shared with 81 genes with tissues and cells affected by HFD exposure; similarly there were shared DEGs seen for AG (48 genes) and SM (32 genes), the conditions that shared most DEGs with CMP. To clarify the biological relevance of the identified DEGs, GO and molecular pathways analysis was performed which revealed pathways with significantly altered activity. Among such pathways, MAPK signalling cascades have been reported to be prominent in the pathogenesis of cardiac and vascular disease [61, 62, 63]. Another pathway, RAS signalling, plays a critical role in cardiac hypertrophy, which suggests complexity in developing meaningful therapy for individuals with these RASopathies [64]. Clinical and genetic studies have also revealed close relationships between cell adhesion proteins and the occurrence of various CMPs [65], thus indicating the important role of focal adhesion pathways in CMP. Related to this, extracellular matrix alterations may be a significant factor in the pathogenesis of dilative CMP [66]. Moreover, molecular pathways hypertrophic CMP, arrhythmogenic right ventricular CMP, dilated CMP pathways were notably and consistently seen to be enriched in CMP.

Analysis of PPIs can provide some detailed insights into the central mechanism behind the diseases [9, 11, 12]. Therefore, we reconstructed the PPI networks around the protein encoded by the DEGs. Based on our topological analysis, we detected ten hub proteins (CDK2, ATM, CDT1, NCOR2, HIST1H4A, HIST1H4B, HIST1H4C, HIST1H4D, HIST1H4E and HIST1H4L) involved in the CMP. A brief description of hub proteins, their gene ontology and features are presented in Table 4. Among the hubs, CDK2 involved in the regulation of myocardial ischaemia and reperfusion injury [67, 74]. The hub protein apical transverse motion (ATM) is associated with electromechanical dyssynchrony in adult dilated CMP [68]. The ATM protein involved in CMP associated with obesity and IR [75]. The hub protein CDT1 is associated with genotoxic stress, which results in aberrant cell proliferation leading to cancer formation [69], but its association with the CMP is not known. Another hub protein, NCOR2 has been reported to be associated with non‐alcoholic fatty liver disease [70], which is one of the prominent risk factors for cardiovascular disease. Yin *et al.* [72] have reported the dysregulation of HIST1H4B in rat cardiomyocytes. The other hub proteins, HIST1H4A, HIST1H4D, HIST1H4E and HIST1H4L were not reported to CMP yet. These identified hubs proteins might be considered as candidate biomarkers or, if their biological role is confirmed, as potential drug targets.

**Table 4:** Summary of hub proteins identified from PPI network

Symbol	Description	Gene ontology	Feature
CDK2	Cyclin Dependent Kinase 2	transferase activity	involved in the regulation of myocardial ischaemia and reperfusion injury [67]
ATM	ATM Serine/Threonine Kinase	transferase activity	associated with electromechanical dyssynchrony in adult dilated CMP [68]
CDT1	Chromatin Licensing and DNA Replication Factor 1	—	genotoxic stress which result in aberrant cell proliferation [69]
NCOR2	Nuclear Receptor Corepressor 2	sequence‐specific DNA binding	associated with non‐alcoholic fatty liver disease [70]
HIST1H4A	Histone Cluster 1 H4 Family Member A	histone binding	implicated a strong involvement of inflammatory‐immune pathways [71]
HIST1H4B	Histone Cluster 1 H4 Family Member B	histone binding	dysregulation of HIST1H4B in rat cardiomyocytes [72]
HIST1H4C	Histone Cluster 1 H4 Family Member C	histone binding	hyperinsulinemic hypoglycemia familial 1 [73]
HIST1H4D	Histone Cluster 1 H4 Family Member D	histone binding	involved in meiosis and signalling pathways by Rho GTPases [73]
HIST1H4E	Histone Cluster 1 H4 Family Member E	histone binding	involved in meiosis and signalling pathways by Rho GTPases [73]
HIST1H4L	Histone Cluster 1 H4 Family Member L	histone binding	involved in meiosis and signalling pathways by Rho GTPases [73]

Based on the network‐based approach, our analyses revealed novel relationships between CMP and other susceptibility/causative factors. This study identified potential biomarkers, which may be candidates for the development of prognostic strategies and treatments. Since the common pathways may indicate ways by the risk factors influence CMP, such pathways and their hub genes identified in this study may have important pathogenic roles in CMP. To examine this and so to validate the results of this systems biology approach, we also analysed the DEGs associated with CMP and each of the risk factors with OMIM databases and dbGAP databases using the valid gold benchmark the disease‐gene associations (Table 5). The DEGs of nine risk factors were identified as showing suggestive links that may promote CMP development and progression. This analysis furnishes new hypotheses that may point the way to establishing mechanistic links between the CMP and the various risk factors that we examined.

**Table 5:** Gene‐disease association analysis of differentially expressed genes of nine risk factors using OMIM and dbGAP databases for CMP

Risk fac./Causes	Gene	Adjusted P‐value
AG	SGCD, TNNT2, TNNC1, LDB3, TTN	1.85×10^−3^
SM	OPRD1, SMAD1, BUB1, PKP2	2.18×10^−1^
DEP	HNF4A, TSHR	4.18×10^−2^
IR	WWOX, MCTP2, BACH2, ZNF783, PPARA	4.62×10^−1^
RM	HMGA2, ADCY2, MYH6	3.34×10^−1^
AC	VWF	2.42×10^−1^
OB	ABCC9, TFAP2A, DMD, FOS	1.45×10^−1^
HCD	SMAD1, DSC2	1.02×10^−1^
HFD	AKAP13, CREBBP, TGFB3, HNF4A, HMGA2, FMN2, ADAMTS7, TGFB3	2.29×10^−1^

## 5 Conclusion

In this study, the genetic association of CMP with various diseasome was identified from comprehensive transcriptomics analyses incorporated with human biomolecular networks to reveal candidate biomarkers at RNA level (transcripts and miRNAs) and protein levels (hub proteins); identified as potential key signalling and regulatory biomolecules in CMP; we also identified possible molecular pathways with CMP involvement. Protein–drug interaction studies revealed eight gene products that had detectable in silico interaction with four compounds including, Amoxapine, L‐Glutamic Acid, Amitriptyline and Acamprosate, which are all compounds already available for therapeutic use apart from glutamate, with is a nutrient and neurotransmitter. Thus, new gene‐based recommendations for disease diagnosis and possible treatment were demonstrated in this study. The molecular signatures and repurposing of the drugs presented in this study may thus deserve attention for use in further experimental studies of CMP.
